# Machine Learning Frameworks for Wearable-Based Stress Modeling in Naturalistic Settings: Scoping Review

**DOI:** 10.2196/76632

**Published:** 2026-07-31

**Authors:** Shifali Sharma, Aswin Kumar Janakiraman, Lujie Karen Chen

**Affiliations:** 1 Department of Information Systems, University of Maryland, Baltimore County, 1000 Hilltop Cir, Baltimore, MD, United States, 1 412 657 5305

**Keywords:** mental health, stress detection, naturalistic setting, in the wild, field studies, wearables, physiology, machine learning models, scoping review

## Abstract

**Background:**

Stress, as commonly recognized, is an integral part of modern life and can significantly affect both mental and physical health. While substantial advancements have been made in measuring physical fitness through wearable devices, the detection and assessment of mental stress remain in their early stages.

**Objective:**

The objective of this paper is to review recent studies of wearable-based stress detection in naturalistic settings, with a specific focus on characterizing machine learning frameworks inspired by the model card approach.

**Methods:**

This review was conducted using the PRISMA-ScR (Preferred Reporting Items for Systematic Reviews and Meta-Analyses extension for Scoping Reviews) checklist. A total of 353 articles were identified through searches in databases such as PubMed, MEDLINE, ScienceDirect, IEEE, ACM Digital Library, Web of Science, and Embase. Studies were considered eligible if they collected data from healthy adults in naturalistic settings using wearable devices and used machine learning models for stress detection.

**Results:**

A total of 34 articles met the eligibility criteria, including 11 conference papers, 22 journal articles, and 1 preprint published between 2017 and 2024. From these studies, we analyzed key machine learning modeling decisions such as problem formulation, ground truth determination, and machine learning algorithms. Additionally, we examined the major contributions of each study, focusing on the challenges they addressed and the solutions they proposed. Based on these findings, we proposed a model card framework for reporting machine learning–based, wearable-based stress detection.

**Conclusions:**

This scoping review highlights recent trends in machine learning models for stress detection and measurement using wearable signals. It underscores the need for improved standardization in reporting practices for datasets and key machine learning decisions, as well as the importance of addressing critical challenges associated with data collection in real-world settings. We hope this review will support and strengthen ongoing research efforts, promote knowledge sharing, and promote collaboration among researchers—ultimately advancing the field as a community.

## Introduction

Stress affects the well-being of many individuals in modern society [1,2]. From an evolutionary perspective, stress is a fight-or-flight response to perceived threat or danger, which prompts necessary survival actions to flee from dangerous situations [3]. However, sustained unmanaged stress may have long-term effects on both mental and physical health [4]. In extreme conditions, it may lead to serious adverse health outcomes such as depression, cardiovascular diseases [5-7], substance abuse, drugs and alcohol addiction, or self-harm behaviors such as suicide [8]. This underscores the importance of timely interventions or management of stress in daily life. Stress detection is fundamental to this type of support system, which permits continuous monitoring of stressful states in naturalistic settings, that is, everyday life. Given their nonintrusive and user-friendly design [9], wearables have been explored to monitor physical fitness, using metrics such as step counts or heart rates. In recent years, there have been emerging interests in using wearables to understand individuals’ psychological fitness or well-being, in which stress detection and measurement are the major driving components.

Although stress detection from instruments such as wearables is not new, detecting stress in naturalistic settings poses significant new challenges. For studies conducted in controlled settings [10-12] in laboratory settings, researchers prescribe stress-inducing activities that participants engage with, such as the Trier Social Stress Test, the Stroop Color-Word Interference Test, the Montreal Imaging Stress Task, the Cold Pressor Test, and the “Sing-a-Song” Stress Test [13]. Similarly, in studies outside of the laboratory, participants may participate in activities that are known to induce stress for some, such as hackathons, driving, or presentations [14]. In contrast, stress detection in naturalistic settings requires individuals to be involved in normal daily activities, often with significant uncertainty regarding the timing, duration, stressor, and stress responses. Moreover, there is additional complexity in dealing with motion artifacts of wearable signals resulting from ambulatory participants [13]. Both issues need to be appropriately addressed for reliable stress detection models.

The massive amounts of high-resolution data collected from wearables provide opportunities to leverage advanced analytics, machine learning (ML), and AI models for stress detection. In recent years, there has been a growing trend in stress detection research using ML as the primary modeling technique. This trend is supported by wearables data collected unobtrusively in naturalistic, real-world settings, driven in part by the availability of large-scale, open-source datasets that attempt to measure psychological constructs such as stress.

While several literature reviews have evaluated studies on wearable-based stress detection, most have focused on cataloging wearables, including their models, sensor types, and factors such as placement, cost, and usability [1,3,15-17].

Several prior reviews have examined aspects of ML-based stress modeling. For example, Namvari et al [16] focused on classification models, which discussed feature engineering approaches and summarized model performance. However, only 5 of the 23 studies included in their review were conducted exclusively in real-life or naturalistic settings. Similarly, the scoping review by Bolpagni et al [3] evaluated 56 studies, of which only 13 were conducted in real-world contexts. Their review primarily focused on preprocessing pipelines, feature extraction methods, and ML model types. Pinge et al [18] reviewed 39 studies in which stressors were predominantly derived from laboratory-induced or controlled stress-inducing stimuli, such as public speaking tasks or exposure to horror movies. Only a small subset of the reviewed studies involved those from free-living conditions. This review summarized preprocessing strategies, feature computation methods, ML techniques (including both classical and deep learning models), and commonly used performance metrics.

In summary, there are several notable gaps in the wearable-based ML-focused review for stress modeling. (1) None of these reviews focus exclusively on studies using data collected in real-world, naturalistic settings. (2) None of the reviews address methodological rigor explicitly, for example, by evaluating whether models use appropriate experimental setups to avoid data leakage [19-22]. This issue is particularly relevant but easy to overlook in wearable studies with repeated measures from the same participants, and failure to account for it can lead to overestimation of model performance. (3) None of the reviews focus on important problem formulation decisions, such as how to appropriately configure the training dataset so that input and output windows are correctly aligned so that it matches the modeling objectives, for example, whether the goal is to detect stress in the present moment (ie, nowcasting) or predict stress in the future (ie, forecasting). (4) There is no review attempting to explicitly tackle the standardization of reporting, for example, by adopting an existing ML model reporting framework such as model card [23]. This scoping review aims to address these critical gaps.

The main contributions of this paper are as follows:

1. Provided a focused review of recent studies on wearable-based stress detection exclusively in naturalistic settings using ML techniques.
2. Analyzed model performance with a focus on methodological rigor, explicitly examining potential threats to validity (eg, data leakage).
3. Analyzed key problem formulation decisions such as input and output window configuration, and distinctions between nowcasting and forecasting problems.
4. Proposed standardized report ML framework in wearable-based stress detection, inspired by the model card model reporting framework.

This review paper is organized as follows: The “Methods” section describes the process used to select papers for review, including eligibility criteria and the PRISMA (Preferred Reporting Items for Systematic Reviews and Meta-Analyses) diagram. The “Results” section presents the dataset features and ML frameworks used in the selected studies. Finally, the “Discussion” section analyzes the findings, identifies limitations, and provides recommendations for future research.

## Methods

### Overview

We adopt a scoping review approach to systematically identify gaps in the existing literature. Although stress detection has long been studied in laboratory settings, it remains challenging to translate these findings into real-life contexts. As such, our primary goal is to map current research on stress detection, identify existing gaps, and provide an overview of ML frameworks inspired by the concept of model cards. Specifically, this review examines how ML frameworks are used to detect stress in naturalistic settings using wearable sensors. The review is guided by the methodological framework proposed by Arksey and O’Malley [24] and adheres to the PRISMA-ScR (Preferred Reporting Items for Systematic Reviews and Meta-Analyses extension for Scoping Reviews) guidelines (Checklist 1) [25].

### Search Strategy

The primary search for this scoping review was conducted in April 2024 across 7 databases: PubMed, MEDLINE, ScienceDirect, IEEE Xplore, ACM Digital Library, Web of Science, and Embase. These databases were chosen for their extensive coverage of recent physiology research on stress detection. Our aim was to identify articles examining stress detection in naturalistic settings using biosignals and wearables. We restricted our search to English-language, peer-reviewed studies published between January 2017 and April 2024 that were available in full text. Initially, we tested various search strings—for instance, using “stress detection” alone yielded too many results, whereas “stress detection” AND (“naturalistic setting” OR “field study” OR “real life”) yielded too few. Ultimately, using more generic terms such as “stress detection,” “wearables,” and “physiology,” combined with AND operators, produced a sufficiently broad yet focused list of results. Research articles involving either primary or secondary data analyses (or both) were included. Primary data analysis refers to studies where data were collected by the authors, whereas secondary data analysis refers to studies using data collected by other researchers, which were either openly available or accessible upon request.

### Eligibility Criteria

Studies were deemed eligible for inclusion if they used data collected in naturalistic (real-world or in-the-wild) settings. Here, naturalistic refers to situations in which stress is not experimentally induced or linked to known stress-inducing events (eg, taking a test), but instead reflects stress as it occurs in daily life. This criterion applied even if some studies use multiple datasets, including those from controlled or laboratory environments, provided that at least one dataset was collected in the wild. This decision was motivated by the fact that stress labeling mechanisms in laboratory studies differ fundamentally from those used in real-life or naturalistic contexts, where stress must be explicitly self-reported or otherwise captured from participants during their daily lives, which pose unique challenges. Given the study’s focus on acute stress, only research involving healthy populations in everyday contexts was considered. Consequently, the use of wearable devices was a prerequisite, as they facilitate continuous monitoring in daily life. Recognizing the surge in wearable technology adoption in recent years, the review encompassed studies published between 2017 and 2024. The specific eligibility criteria pertinent to this scoping review are detailed in Textbox 1.

Textbox 1. **Inclusion and exclusion criteria.**

**Inclusion criteria**

- Data were collected in naturalistic settings where participants engaged in normal, everyday activities (including work).
- Healthy participants were involved. At least one type of data was collected using wearable devices to capture physiological and/or psychological signals.
- Machine learning models or other advanced data-driven techniques were used to detect stress.
- The study needs to be published in English.

**Exclusion criteria**

- Data were collected only in controlled settings (eg, in laboratory environments or when participants engaged in known stress-inducing activity such as a hackathon as described in the “Introduction”).
- The participants had been diagnosed with mental health disorders or other chronic conditions.
- No wearable devices were used in the data collection process.
- No machine learning or other advanced data-driven modeling techniques were used to detect stress.
- The study was not published in English.

### Title and Abstract Screening

We began by applying basic eligibility filters (language and publication year) within the scientific databases themselves. After eliminating duplicate entries, the first author compiled all the retrieved records into a spreadsheet and conducted the initial title and abstract screening using preagreed eligibility criteria. The third author reviewed and verified the screened records and decisions regarding the inclusion and exclusion in the spreadsheet against the same criteria. During abstract screening, we identified publicly available, on-request datasets containing naturalistic wearable data for stress detection and reviewed their citations, adding any studies that used these datasets to our review. Any ambiguities or discrepancies were resolved through discussion until consensus was reached. Since formal full parallel double-screening was not performed, Cohen κ or percentage agreement was not calculated. Finally, the remaining full-text articles were reassessed for eligibility using the specified inclusion and exclusion criteria.

### Full-Text Review, Data Extraction, and Analysis

Data extraction involved classifying studies into predefined categories to facilitate evidence synthesis, with a particular focus on the design of ML frameworks inspired by model cards. Extracted data elements included availability of datasets such as indicating whether datasets were open source, available upon request, or collected within the study design, demographic information such as participants' professions, total number of participants, and geographic location, details of wearable devices and biosignals used, duration of data collection, primary research focus such as problem addressed which are either originating from domain-centric or data-centric approaches, and ML models used. Additionally, we examined the formulation of problem statements, including the selection of input and output windows for nowcasting or forecasting tasks. We also assessed validation practices, with particular attention to measures implemented to prevent data leakage, such as appropriate data partitioning and cross-validation techniques.

## Results

### Literature Search

**Figure 1.**
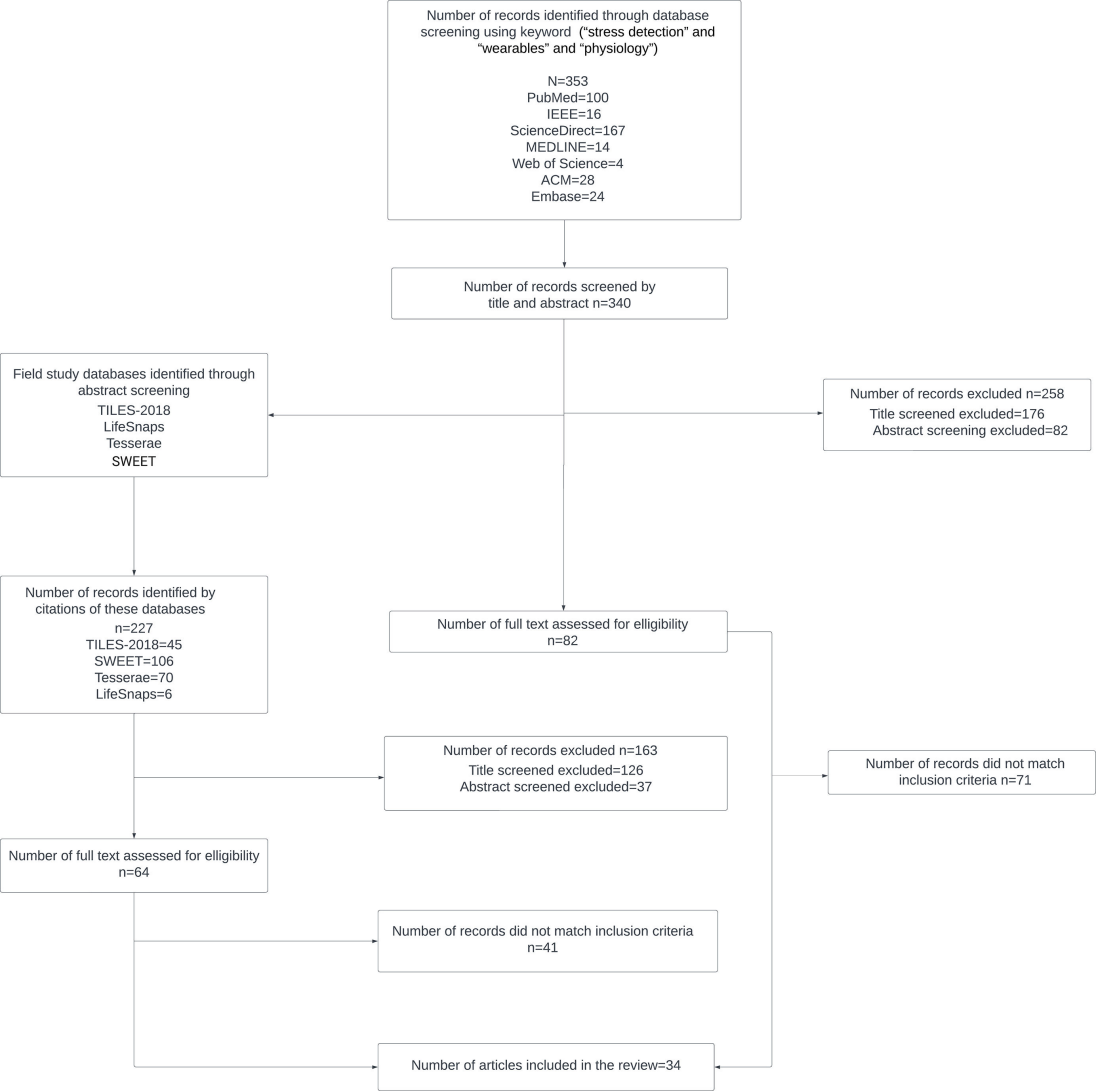
Flowchart for paper screening.

As shown in the flowchart in Figure 1, a total of 353 articles were identified through database searches: 100 from PubMed, 16 from IEEE, 167 from ScienceDirect, 14 from MEDLINE, 4 from Web of Science, 28 from ACM Digital Library, and 24 from Embase. During the title and abstract screening, 258 articles were excluded for not meeting the eligibility criteria.

During the abstract screening phase, we identified additional datasets that were collected using wearables to model wellness-related constructs in naturalistic settings, for example, TILES-2018 [26], Lifesnaps [27], Tesserae [28], and SWEET [29]. In total, we find 227 secondary analysis papers citing those datasets, with 45 of them using TILES-2018, 106 using SWEET, 70 using Tesserae, and 6 using Lifesnaps. After further title and abstract screening, 163 studies were excluded. This left a total of 146 studies considered for full-text review, with a mixture of primary and secondary analyses. Of these, 71 studies were excluded from the primary data analysis category, and 41 studies were excluded from the secondary data analysis category based on the eligibility criteria. In the end, 34 articles were selected for inclusion in this scoping review. For a detailed description of the search and selection strategy using PubMed, please refer to Multimedia Appendix 1.

### Study Characteristics

#### Overview

Among the 34 selected papers, 11 studies were conference proceedings (32%), 22 were journal articles (65%), and 1 was a preprint (3%). The studies were published between 2017 and 2024. Of these, 24 out of 34 (71%) studies conducted secondary data analysis using datasets collected by other researchers—either open-source or available upon request. The remaining 10 out of 34 (29%) studies performed primary data analysis based on datasets collected by the authors specifically for the reported research. Fitbit was the most commonly used wearable device, followed by Garmin, Empatica E4, and OMsignal garments. The duration of data collection across studies ranged from a single day to several months.

#### Dataset Characteristics

Although the dataset is not the primary focus of this review, we provide a brief overview of the dataset characteristics since it forms the foundation for the ML models. A comprehensive overview of the datasets used in the included studies is presented in Table 1. For detailed field descriptions, please refer to Multimedia Appendix 2. The frequently-used datasets as part of the secondary data analysis are TILES-2018, SWEET, LifeSnaps, and Tesserae. The populations represented include clinical providers, office and information workers, police officers, military personnel, university students, and remote workers, while a few studies did not specify the population. The number of participants in the datasets ranged from as few as 3 to as many as 1002. The duration of data collection varied, ranging from 1 day to 10 weeks.

**Table 1.** Dataset characteristics.

Dataset citation key	Dataset name	Participant pool	Number of participants	Data collection duration	Wearable device used
Mundnich et al (2020) [26]	TILES-2018	Clinical providers	212	10 weeks	Fitbit Charge 2 and OMsignal smart garment
Smets et al (2018) [29]	SWEET	Staff members in technology-oriented, banking, and public sector companies.	1002	5 days	imec’s Chillband and chest patch
Yfantidou et al (2022) [27]	Lifesnaps	Participants were recruited through university mailing lists, and although not explicitly stated, they were likely university students from Greece, Cyprus, Italy, and Sweden.	71	4 months (2 waves, each lasting 2 months)	Fitbit Sense
Mattingly et al (2019) [28]	Tesserae	Information workers	757	56 days	Garmin Vivosmart 3, a waterproof wristwatch
Boateng and Kotz (2016) [30]	—^a^	Unknown	10	1 day	Zephyr chest strap and Amulet
Tervonen et al (2020) [31]	—	Office workers	74	4 weeks	Polar M600 smartwatch
Bavaresco et al (2020) [32]	—	Unknown	5	8 days	Polar H7 chest strap
Gjoreski et al (2017) [19]	—	Unknown	5	11 days, on average	Empatica E3 and E4 wrist devices
de Vries et al (2022) [33]	—	Dutch police officers	8	At least 15 weeks, up to 55 weeks.	Oura Ring (Generation 2)
Han et al (2020) [20]	—	Unknown	3	2 days	Empatica E4 wristband and Shimmer3 ECG chest strap
de Vries et al (2023) [34]	—	Dutch military	73	8 weeks	Garmin Tactix Charlie, smartwatch
Tump et al (2022) [35]	—	Remote-working employees of the global insurance company Cigna in 3 locations: the United States, the United Kingdom, and Hong Kong.	198	7 consecutive days	Garmin Vivosmart 4
Mishra et al (2020) [36]	—	University students	26	3 days	Polar H7 chest strap, Amulet wrist device, and a custom GSR^b^ sensor
Schmidt et al (2019) [37]	—	University students	11	Approximately 16 days	Empatica E4 wristband

a Not available.

b GSR: Galvanic skin response.

#### Wearables Characteristic

The choice of wearables has a direct impact on the quality and diversity of data collected. A wide range of wearables has been used in these studies to unobtrusively collect data, with the most frequently used devices being the Garmin Vivosmart [22,35,38,39], Fitbit Charge 2 [21,40], OmSignal garment [41,42], Empatica E4 [20,37], Unihertz Jelly Pro smartphone [41,43], and the Imec Chillband [44]. Wearable sensors enable the capture of diverse data types across multiple modalities, including physiological data, for example, heart rate, temperature, and skin conductance [19,32,33,37,40,45-47]. Additional data types were also recorded, for example, phone usage, sleep duration, circadian rhythm, and movement [21,35,40,48-50], audio signals [21,40,41,49], sociodemographic data [47,50], and environmental data (eg, temperature and humidity) [51]. These modalities provide options for studies to use either unimodal [20,30,36] or multimodal approaches [49,52,53].

#### ML Framework and Model Performances

In this section, we organize the analysis around key methodological decisions identified across the reviewed studies on wearable-based stress detection in naturalistic settings. At a high level, this task was framed as learning a mapping between the physiological signals collected from wearables and the psychological construct of stress. This was often formulated as a supervised learning framework (either a classification or regression problem), where the inputs are wearable sensing data and the outputs are stress labels.

Due to the nature of the data being collected, the reviewed studies varied across several methodological dimensions. We analyze the model frameworks along the following dimensions:

1. Ground truth stress labels: how the labels are collected and the types of labels collected.
2. Input and output windows: how to set up input-output pairs based on corresponding time windows and how the temporal relationships between input and output windows are informed by the problem being addressed—whether it involves in-the-moment stress detection (ie, nowcasting) or predicting future stress (ie, forecasting).
3. ML experiment design: how to appropriately set up ML experiments, for example, split training and testing data appropriately to avoid data leakage.
4. ML models: the primary ML algorithms used in the studies.
5. Challenges and solutions: the specific challenges that the reviewed papers aim to tackle and the main solutions proposed.
6. Analysis of model performance: analysis of model performance with respect to ML and specific solutions to address challenges, with caveats resulting from issues in reporting and methodological rigor.

#### Overview of Model Characteristics

Across the 34 included studies [19-22,30-59], the large majority adopted a global, population-level modeling approach, with 28 [19-22,30,32-40,42,44,46,48-50,52-59] out of 34 (82%) studies following this approach, while only 6 [31,41,43,45,47,51] out of 34 (18%) studies pursued personalization or built individualized models. With respect to model family, 21 [19,20,22,30-36,39,40,42,44,45,47,49,51,53,54,58] out of 34 (62%) studies used traditional ML, while 8 [21,37,41,43,46,48,56,57] out of 34 (24%) studies relied exclusively on deep learning. A further 4 [38,50,52,55] out of 34 (12%) studies combined traditional ML and deep learning, and 1 [59] out of 34 (3%) studies additionally incorporated a large language model (LLM), bringing the total share of studies that used deep learning in some capacity to 13 [21,37,38,41,43,46,48,50,52,55-57,59] out of 34 (38%) studies. Reporting of evaluation metrics was assessed among the 22 classification studies [19-21,30-32,35-38,42,44-49,52,55-58]. *F*_1_-score was the most commonly reported metric, appearing in 19 [19,21,31,32,35-38,42,44-46,48,49,52,55-58] out of 22 (86%) classification studies, followed by accuracy in 12 [20,21,30-32,38,44,46,47,56-58] out of 22 (55%) studies, precision in 7 [19,32,36,38,52,57,58] out of 22 (32%) studies, and recall in 5 [32,36,38,57,58] out of 22 (23%) studies. Only 3 [42,52,55] out of 22 (14%) studies reported alternative discrimination or balance-aware metrics such as area under the receiver operating characteristic curve (AUC), balanced accuracy, or Matthews correlation coefficient (MCC), suggesting limited adoption of metrics that are robust to class imbalance. Among the 13 regression studies [22,32,34,38-41,43,50,51,53,54,59], all reported at least one quality measure such as R-squared or mean-squared error (MSE). Potential data leakage was identified in roughly half of the corpus, with 19 [30,32-35,38,39,41,42,44,45,47,49,53-56,58,59] out of 34 (56%) studies judged to have at least one source of potential leakage and the remaining 15 [19-22,31,36,37,40,43,46,48,50-52,57] out of 34 (44%) studies judged to be free of obvious leakage. Finally, in terms of temporal problem formulation, the field is dominated by nowcasting, with 27 [19-22,30-39,42-44,46,48,49,51-56,58] out of 34 (79%) studies framing their task as inferring a current or concurrent state; only 4 [40,47,50,57] out of 34 (12%) studies explicitly framed the task as forecasting a future state, and the formulation could not be unambiguously determined for 3 [41,45,59] out of 34 (9%) studies.

Details of the key components of the ML framework are presented in Tables 2 and 3, which summarize the reported best model performance; please refer to Multimedia Appendix 3 for additional details.

**Table 2.** Machine learning framework characteristics.

Paper citation key	Dataset citation key	Main problems addressed	Problem formulation	Input window	Output window	Model type	Sample size (N)	Possible data leakage
Boateng and Kotz 2016 [30]	Boateng and Kotz (2016) [30]	No specific problem was addressed	Nowcasting	1 minute or 5 minutes	15 minutes	Classification	10	Yes
Parousidou et al 2023 [45]	Yfantidou et al (2022) [27]	Personalization	Unclear	Unclear	Unclear	Classification	71	Yes
Tervonen et al 2020 [31]	Tervonen et al (2020) [31]	Personalization	Nowcasting	Current day	Current day	Classification	74	No
Bavaresco et al 2020 [32]	Bavaresco et al (2020) [32]	Context awareness	Nowcasting	20 minutes	20 minutes	Classification	5	Yes
Gjoreski et al 2017 [19]	Gjoreski et al (2017) [19]	Context awareness	Nowcasting	A range of windows from 10 minutes to 30 minutes	20 minutes	Classification	5	No
de Vries et al 2022 [33]	de Vries et al (2022) [33]	Temporal dependency	Nowcasting	Previous day	Current day	Regression (statistical)	8	Yes
Han et al 2020 [20]	Han et al (2020) [20]	No specific problem was addressed	Nowcasting	Current day	Current day	Classification	17	No
de Vries et al 2023 [34]	de Vries et al (2023) [34]	Temporal dependency	Nowcasting	Last night’s sleep duration	at wake up	Regression (statistical)	73	Yes
Tump et al 2022 [35]	Tump et al (2022) [35]	Context awareness	Nowcasting	3 hours	3 hours	Classification	198	Yes
Mishra et al 2020 [36]	Mishra et al (2020) [36]	No specific problem was addressed	Nowcasting	1 minute	1 minute	Classification	27	No
Schmidt et al 2019 [37]	Schmidt et al (2019) [37]	No specific problem was addressed	Nowcasting	1 minute	1 minute	Classification	12	No
Jiang et al 2023 [54]	Mundnich et al (2020) [26]	Addressing limited supply of training data	Nowcasting	3 days centered on current day	Current day	Regression	212	Yes
Hadjiantonis et al 2020 [40]	Mundnich et al (2020) [26]	Temporal dependency	Forecasting	Previous day	Current day	Regression (statistical)	130	No
Gaballah et al 2021 [21]	Mundnich et al (2020) [26]	Temporal dependency	Nowcasting	Current shift	Current shift	Classification	212	No
Yu and Sano 2022 [48]	Mundnich et al (2020) [26]	Addressing limited supply of training data	Nowcasting	2.5 hours after the label was collected	Current shift	Classification	212	No
Burghardt et al 2021 [52]	Mundnich et al (2020) [26]	Temporal dependency	Nowcasting	3 days centered on the current day	Current day	Classification	212	No
Kao et al 2020 [51]	Mundnich et al (2020) [26]	Personalization	Nowcasting	3 days centered on the current day	Current day	Regression	212	No
Paromita et al 2023 [43]	Mundnich et al (2020) [26]	Personalization	Nowcasting	Current day	Current day	Regression	212	No
Pimentel et al 2021 [55]	Mundnich et al (2020) [26]	No specific problem was addressed	Nowcasting	Current day	Current day	Classification	212	Yes
Yang et al 2022 [46]	Mundnich et al (2020) [26]	Multiple modalities	Nowcasting	2 hours before the label was collected	Current shift	Classification	212	No
Zanna et al 2022 [56]	Mundnich et al (2020) [26]	Bias mitigation	Nowcasting	2 hours before the label was collected	Current shift	Classification	212	Yes
Tiwari and Falk 2021 [42]	Mundnich et al (2020) [26]	No specific problem was addressed	Nowcasting	Current day	Current day	Classification	212	Yes
Feng and Narayanan 2022 [49]	Mundnich et al (2020) [26]	Context awareness	Nowcasting	Current day	Current day	Classification (stable)	99	Yes
Feng et al 2021 [53]	Mundnich et al (2020) [26]	Context awareness	Nowcasting	Current day	Current day	Regression (stable, statistical)	113	Yes
Ravuri et al 2020 [41]	Mundnich et al (2020) [26]	Personalization	Unclear	Not given	Not given	Regression	154	Yes
Stojchevska et al 2022 [44]	Smets et al (2018) [29]	Context awareness	Nowcasting	1 hour	1 hour	Classification	1002	Yes
Booth et al 2022 [38]	Mattingly et al (2019) [28]	Temporal dependency	Nowcasting	Current day	Current day	Regression and classification	606	Yes
Martinez et al 2022 [39]	Mattingly et al (2019) [28]	No specific problem was addressed	Nowcasting	A range of windows from 5 minutes to 24 hours	Current day	Regression (statistical)	657	Yes
Robles-Granda et al 2021 [22]	Mattingly et al (2019) [28]	Temporal dependency	Nowcasting	Current day	Current day	Regression (stable)	757	No
Saylam and İncel 2023 [50]	Mattingly et al (2019) [28]	Temporal dependency	Forecasting	A range of windows from 1 day to 30 days before	A range of windows from 1 day to 7 days ahead	Regression	757	No
Li et al 2024 [57]	Mattingly et al (2019) [28]	Temporal dependency	Forecasting	A range of windows from 25 days to 35 days before	A range of windows from 12 to 19 days ahead	Classification	478	No
Saylam and Durmaz İncel 2022 [58]	Mattingly et al (2019) [28]	Context awareness	Nowcasting	Current day	Current day	Classification	757	Yes
Kim et al 2024 [59]	Yfantidou et al (2022) [27]	Use of LLM^a^ in stress detection	Unclear	Unclear	Unclear	Regression	16	Yes
Paraschou et al 2023 [47]	Yfantidou et al (2022) [27]	No specific problem was addressed	Forecasting	Unclear	Unclear	Classification	71	Yes

a LLM: large language model.

**Table 3.** Machine learning model performance.

Paper citation key	Classification model					Regression model	Baseline model reported	Uncertainty measure reported
	Accuracy	*F*_1_-score	Precision	Recall	Other measures	Quality measure		
Boateng and Kotz 2016 [30]	1.00	—^a^	—	—	—	N/A^b^	No	No
Parousidou et al 2023 [45]	—	0.66	—	—	—	N/A	Yes	No
Tervonen et al 2020 [31]	0.51	0.62	—	—	—	N/A	Yes	Yes
Bavaresco et al 2020 [32]	0.82	0.75	0.9	0.64	—	N/A	No	No
Gjoreski 2017 et al [19]	—	0.9	0.95	—	—	N/A	No	No
de Vries et al 2022 [33]	N/A	N/A	N/A	N/A	N/A	Adjusted *R*^2^: 0.01‐0.23	N/A	N/A
Han et al 2020 [20]	1.00	—	—	—	—	N/A	No	No
de Vries et al 2023 [34]	N/A	N/A	N/A	N/A	N/A	Marginal *R*^2^=0.004	N/A	N/A
Tump et al 2022 [35]	—	0.47	—	—	—	N/A	Yes	Yes
Mishra et al 2020 [36]	—	0.7	0.57	0.91	—	N/A	No	No
Schmidt et al 2019 [37]	—	0.47	—	—	—	N/A	Yes	Yes
Jiang et al 2023 [54]	N/A	N/A	N/A	N/A	N/A	MSE^c^~1.00	Yes	Yes
Hadjiantonis et al 2020 [40]	N/A	N/A	N/A	N/A	N/A	*r*=0.24 (*P*<.01)	Yes	No
Gaballah et al 2021 [21]	0.66	0.64	—	—	—	N/A	No	No
Yu and Sano 2022 [48]	—	0.70	—	—	—	N/A	Yes	Yes
Burghardt et al 2021 [52]	—	0.23	0.16	—	AUC^d^=0.56	N/A	Yes	No
Kao et al 2020 [51]	N/A	N/A	N/A	N/A	N/A	RMSE^e^=0.89; *r*=0.42; *R*^2^=0.15	Yes	No
Paromita et al 2023 [43]	N/A	N/A	N/A	N/A	N/A	One-tailed paired *t* test (*P*<.001)	No	Yes
Pimentel et al 2021 [55]	—	0.68	—	—	BACC^f^=0.65; MCC^g^=0.30	N/A	No	Yes
Yang et al 2022 [46]	0.58	0.72	—	—	—	N/A	No	No
Zanna et al 2022 [56]	0.43‐0.54	0.30‐0.42	—	—	—	N/A	No	No
Tiwari and Falk 2021 [42]		0.69	—	—	BACC=0.66; MCC=0.31	N/A	No	Yes
Feng and Narayanan 2022 [49]	—	66	—	—	—	N/A	No	No
Feng et al 2021 [53]	N/A	N/A	N/A	N/A	N/A	Adjusted *R*^2^: (PA^h^ 0.152; NA^i^ 0.034)	N/A	N/A
Ravuri et al 2020 [41]	N/A	N/A	N/A	N/A	N/A	ρ=0.08	No	Yes
Stojchevska et al 2022 [44]	0.42	41	—	—	—	N/A	No	Yes
Booth et al 2022 [38]	0.62	0.75	0.65	0.89	—	ρ=0.25	Yes	Yes
Martinez et al 2022 [39]	N/A	N/A	N/A	N/A	N/A	Marginal *R*^2^: 0.022 (main); 0.032 (follow-up)	N/A	N/A
Robles-Granda et al 2021 [22]	N/A	N/A	N/A	N/A	N/A	SMAPE^j^=0.66	Yes	Yes
Saylam and İncel 2023 [50]	N/A	N/A	N/A	N/A	N/A	MAE^k^=0.47	No	No
Li et al 2024 [57]	0.74	72	0.73	0.71	—	N/A	No	No
Saylam and Durmaz İncel 2022 [58]	0.85	0.85	0.85	0.85	—	N/A	No	No
Kim et al 2024 [59]	N/A	N/A	N/A	N/A	N/A	MAE=0.32	No	Yes
Paraschou et al 2023 [47]	0.92	—	—	—	—	N/A	No	No

a Not available/not reported.

b N/A: not applicable.

c MSE: mean-squared error.

d AUC: area under the receiver operating characteristic curve.

e RMSE: root mean square error.

f BACC: balanced accuracy.

g MCC: Matthews correlation coefficient.

h PA: positive affect.

i NA: negative affect.

j SMAPE: symmetric mean absolute percentage error.

k MAE: mean absolute error.

#### Ground Truth Stress Labels

The goal of ML models is to predict or estimate psychological constructs, such as stress [43]. These constructs are often referred to as ground truth labels or targets within an ML framework. Since data are collected in naturalistic settings, aligning stress labels with physiological data from wearables poses a challenge, as there are no obvious ground truth labels, unlike in controlled environments where the stressor and stress-inducing periods are known with high levels of certainty. In naturalistic settings, stress labels are typically collected from participants through self-report mechanisms, such as ecological momentary assessment (EMA) [45]. EMA involves short surveys delivered through phone-based apps, a method used by most of the studies. In this section, we outline several key decisions involved in collecting ground truth labels using EMA.

### EMA Prompts Frequency and Delivery Mechanisms

EMA was most frequently administered daily [48,51,54], capturing a range of psychological constructs. In fewer instances, more frequent assessments were conducted (eg, every 30 minutes or every 2 hours throughout the day) [30-32]. In all studies, EMA prompts were triggered by predefined schedules, with most being delivered via mobile apps, often through custom-developed apps [21,48,51]. A smaller number of studies used SMS messages to deliver EMA surveys [38,39,50].

### Response Window

Since the data were collected in real-world contexts, participants were not expected to respond promptly to EMA probes as they may have been busy with other tasks. To control the delay in response and ensure the accurate timing of the label, studies may put an upper bound on the delay, that is, the probe will expire after a certain period of time [32] to ensure the relevance of labels.

### Types of Stress Labels and Instruments Used

The most commonly collected psychology constructs in the studies were stress, which occurred in 26 [19-21,30-39,41-48,50,51,55,57-59] out of 34 (76%) studies, anxiety in 9 [22,39,43,50,51,53,55,56,59] out of 34 (26%) studies, positive or negative affect in 9 [22,39,41,43,49-51,53,54] out of 34 (26%) studies. Various instruments were used, including the Short State-Trait Anxiety Inventory (S-STAI) [47,59], which assessed both stress and anxiety, and the Perceived Stress Scale (PSS) [21,48]. Anxiety was also measured using the State-Trait Anxiety Inventory (STAI) [42,56]. The Positive and Negative Affect Schedule (PANAS) short scale [49,53] was used to assess both positive and negative effects.

### Stress Measurement Scale

Additionally, different scales were used, such as the Likert scale [31,36,37], Numeric Rating Scale [33], and Visual Analog Scale [35], which is a gliding scale ranging from “not at all” to “extremely.” In a few studies, discrete ordered scales were used to categorize stress levels as low, medium, or high [30,58].

### Preprocessing of Stress Labels

There were various preprocessing decisions researchers have made with regard to the stress labels in the reviewed studies. In most studies, the output targets in the ML model maintained the same granularity as the ground truth stress label. However, some studies applied downsampling techniques. For instance, when ground truth labels were collected every 30 minutes, they were aggregated into daily values [20,31] in modeling.

In classification studies, these outputs collected on a numeric scale were often categorized into bins or classes, such as low, medium, or high levels of stress or anxiety [19]. Most studies used binarized output targets for classification, while only a few applied multiclass classification. Due to the ordinal nature of the data, multiple categories were combined into fewer groups. For example, on a 5-point scale, ratings of 0‐1 were classified as stress, while ratings of 2‐4 were classified as no stress [19,32]. Another example involves merging the last 3 categories of a 5-point scale into a single “high stress” group, with the first 2 categories remaining as “low” and “medium stress” [44]. For nondiscrete values, such as on a 100-point scale, values below 50 were marked as low stress, and values above 50 were marked as high stress [35].

Two primary methods were used to define classification label thresholds: a personalized approach and a global approach. The personalized approach involved setting thresholds specific to individual participants, whereas the global approach applied the same thresholds across all participants. For personalized thresholds, various statistical methods were used, including majority voting, median calculation, and z-score analysis. The z-score method was the most commonly used [21,46,48,56], where z-scores were calculated for each individual to account for subjective variability. Data were then categorized into 2 classes; for example, negative classes included those with z-scores below zero, and vice versa. In the global approach, values were discretized using predefined scales or thresholds derived from the entire participant sample. For instance, some studies applied fixed global thresholds to classify stress and anxiety levels across all participants [42].

### Input and Output Windows

As part of the modeling framework, we extracted the input and output windows reported in the reviewed studies. The input window refers to the time frame from which input features are extracted, typically derived from physiological and behavioral signals. On the other hand, the output window defines the period during which the model predicts target outcomes corresponding to the ground truth labels, as discussed in the “Ground Truth Stress Labels” section.

**Figure 2.**
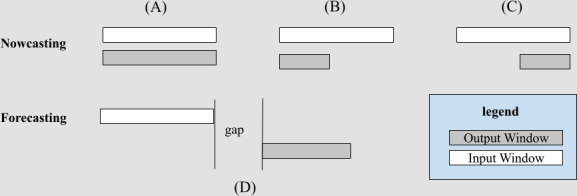
The relationship between input and output windows; nowcasting versus forecasting.

We use the terms nowcasting and forecasting to describe the relationship between input and output windows. Nowcasting (Figure 2A-C) refers to models that predict outcomes, such as stress, for the present or immediate future, resulting in a very short output window with no gap between input and output windows. In contrast, forecasting (Figure 2D) predicts stress over future time periods, with short-term forecasting focusing on the immediate next period and long-term forecasting predicting stress at a time point extended further into the future.

Several studies have used an exact overlap between input and output windows as shown in Figure 2A, often with a window size of 24 hours [31,51]. A smaller number of studies have used shorter input-output windows, such as every 20 minutes [32], hourly [44], or every 3 hours [35]. This exact overlap is a defining characteristic of nowcasting studies, as illustrated in Figure 1.

Additionally, many studies included partial overlap, where the input window is either longer or shorter than the output window, as shown in Figure 2B and C. For example, an input window might include data from the previous day, the current day, and the next day, while the output window focuses only on the current day [51,52] or both the previous and current days [54]. In other examples, input window is shorter than the output window; for example, the input window covers 1 minute and the output window spans 15 minutes [30], or where the input window is 2 hours paired with a 1-day output window [56], or an input window that selects an optimal range between 10 and 27.5 minutes while the output window remains fixed at 20 minutes [19].

In the forecasting studies identified, the input window is chosen to be immediately adjacent and trail the output window (Figure 2D). For example, the input window may consist of data from the previous day, with the output window set for the current day [40], in which case the gap is zero. In other instances, the input window might consist of data from the last 35, 30, or 25 days, while the output window could be set for 19, 17, or 12 days [57] into the future, starting from the prediction time, in which case the gap is larger than zero.

**Figure 3.**
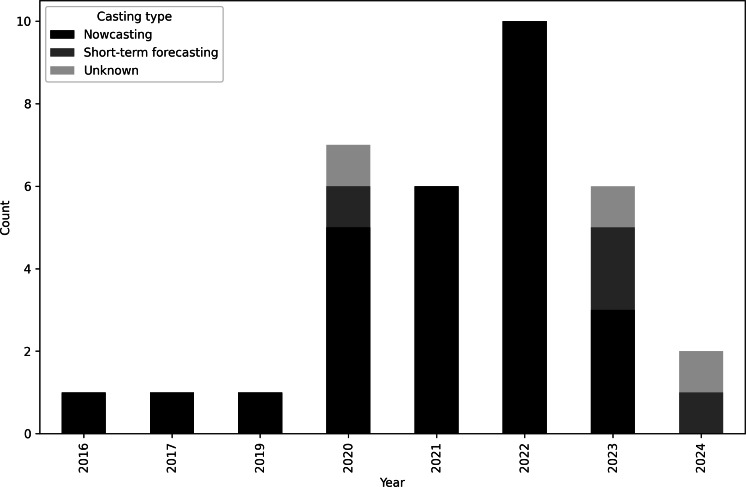
Number of studies formulated as nowcasting versus forecasting problem, grouped by year.

It has been observed from the selected studies that the majority of researchers have used a nowcasting relationship between the input and output windows, as illustrated in Figure 3.

### Addressing Issues of Data Leakage

The reviewed studies differed in how their training, test, and validation sets were split. Since the wearable dataset is longitudinally collected, it is typical to see multiple input-output pairs extracted from the same participants. Data leakage can occur where data points in the training set and test set may originate from the same participant. This made participant-level splitting relevant to the assessment of potential data leakage and possible inflation of reported model performance.

In our review, we observed that 13 [30,32,35,36,41,42,45,47,54-56,58,59] out of 28 applicable (46%) studies [19-21,30-32,35-38,40-48,50-52,54-59] did not explicitly address potential data leakage issues as inferred from the description of the split of training, test, or validation set, while the rest addressed data leakage issues by using strategies such as leave-one-subject-out cross-validation.

### ML Models

This section provides insights into the types of ML models used. The studies included in this review primarily focused on supervised learning paradigms, addressing both classification and regression tasks. A diverse range of models was used, spanning traditional ML frameworks to end-to-end deep learning architectures. For feature extraction, most studies relied on statistical summaries [30,31,36], while a smaller subset applied Fourier transformations, particularly for analyzing heart rate variability signals [19,20,42].

Traditional ML algorithms, valued for their simplicity and interpretability, such as support vector machines (SVMs), Naive Bayes, and k-nearest neighbors (KNN), were frequently used. Additionally, various ensemble methods, including bagging and boosting techniques, were widely applied [19,44,45,47,51]. Among these, random forest (RF) was the most commonly used in studies using traditional ML algorithms [19,36,38,44,45,49,50,52,54,58,59].

Table 4 summarizes the most frequently used traditional ML models across the studies.

**Table 4.** Counts of papers using specific traditional machine learning models.

Model	Count of papers using the model	Reference numbers
Random forest	11	Parousidou et al 2023 [45], Gjoreski et al 2017 [19], Mishra et al 2020 [36], Jiang et al 2023 [54], Burghardt et al 2021 [52], Feng and Narayanan 2022 [49], Booth et al 2022 [38], Robles-Granda et al 2021 [22], Saylam and İncel 2023 [50], Saylam and Durmaz İncel 2022 [58], Kim et al 2024 [59]
SVM^a^	9	Boateng and Kotz 2016 [30], Bavaresco et al 2020 [32], Gjoreski et al 2017 [19], Han et al 2020 [20], Mishra et al 2020 [36], Burghardt et al 2021 [52], Pimentel et al 2021 [55], Tiwari and Falk 2021 [42], Kim et al 2024 [59]
Boosting methods	7	Gjoreski et al 2017 [19], Stojchevska et al 2022 [44], Parousidou et al 2023 [45], Paraschou et al 2023 [47], Saylam and İncel 2023 [50], Kao et al 2020 [51], Jiang et al 2023 [54]
Naive Bayes	4	Parousidou et al 2023 [45], Bavaresco et al 2020 [32], Gjoreski et al 2017 [19], Han et al 2020 [20]
KNN^b^	4	Bavaresco et al 2020 [32], Gjoreski et al 2017 [19], Han et al 2020 [20], Jiang et al 2023 [54]
Logistic regression	4	Parousidou et al 2023 [45], Tump et al 2022 [35], Jiang et al 2023 [54], Burghardt et al 2021 [52]
Decision tree	3	Parousidou et al 2023 [45], Gjoreski et al 2017 [19], Robles-Granda et al 2021 [22]

a SVM: support vector machine.

b KNN: k-nearest neighbors.

In addition to traditional ML, the use of deep learning models has seen a significant rise. Models explored include recurrent neural networks (RNNs) and long short-term memory (LSTM) networks, convolutional neural networks (CNNs), and LLMs. The choice of models is closely tied to the specific objectives and implications of each study, as discussed below.

### Challenges and Solutions

The reviewed studies aimed to address specific challenges, each accompanied by proposed solutions. These challenges were either intrinsic to the data or inherent to the domain. Some common challenges and their corresponding solutions are outlined below

### Personalization

Personalization was one of the challenges addressed in the reviewed studies, because of intrinsic between-person variability, as individuals may respond differently to the same stressors. In many of the selected studies, personalization was not considered, thus following a one-size-fits-all approach in modeling. However, a notable number of studies addressed personalization as part of the preprocessing phase. Two key personalization approaches were identified: user-based and group-based. While the user-based approach involves creating a unique model for each participant, the group-based approach groups participants based on selected attributes, such as demographics or gender.

Various clustering algorithms were used in the studies to create cohorts or groups, including k-means, spectral clustering, agglomerative clustering, mean shift, affinity propagation, and Gaussian mixture models (GMM) [51]. Some other studies also used additional techniques to enhance cluster quality, such as iterative clustering [41], which progressively refined participant groupings by repeating the clustering process multiple times. Another method listed in the reviewed studies was dimensionality reduction prior to clustering, which was achieved through principal component analysis (PCA) [43]. PCA simplified the feature space while preserving variance by projecting the data into a lower-dimensional space, whereas self-organizing maps (SOMs) [31] facilitated the organization of complex patterns within the data.

The clusters created during the preprocessing step were further trained using traditional ML techniques (eg, RF, decision trees, and linear regression) [45] as well as deep learning approaches, including metric learning with Siamese neural networks (SNNs) [43]. SNNs transform input data to ensure that similar data points are projected close together, while dissimilar points are spaced apart in the learned feature space. Additionally, feedforward neural networks (FFNs) [41] were used to fine-tune the generalized model by adjusting its weights through iterative training on group-specific data.

Clustering offers several benefits. For example, it may induce more cohesive groups as grouping users with similar characteristics helps the model detect common stress patterns within each group [45]. In another study, the user-based collaborative filtering method is used by leveraging health information from other users. This type of model can fill gaps within groups and reduce the reliance on historical data from individual users [51].

### Temporal Dependency

Data collected from wearable sensors inherently possess a time-dependent nature due to its intrinsic properties. Consequently, studies have explored methods to explicitly address these temporal dependencies to improve model accuracy, with the hope that those methods may reveal complex relationships that conventional ML models may overlook. Various models specifically designed to capture these temporal dependencies are listed below, along with a brief summary of their usage in the selected studies.

1. LSTM networks, designed to capture long-term dependencies in sequential data, were used as bidirectional LSTM (BI-LSTM) in one of the studies [21]. In that study, BI-LSTM captured both past and future values to predict current stress levels in a nowcasting setup. An hourly vector was processed in both the forward and backward directions to analyze data from start to finish and vice versa. The outputs from both directions were combined to generate the final predictions, offering a comprehensive understanding of the hourly temporal context. In another study, a lagged version of the data was explored to highlight trends over time, associating each data point with its historical values, allowing the model to learn from past trends [38].
2. Vector autoregression (VAR) [33] was used to predict current values based on past data through lag selection. Two approaches were taken: predicting sensor outcomes (eg, total sleep time and heart rate variability) using EMA variables such as stress, and predicting self-reported EMA variables based on sensor data. An impulse response function (IRF) was also applied to visualize how changes in predictor variables affect outcomes over time.
3. A hidden Markov model was used to capture how different physiological conditions evolve over time through hidden states. This study [52] aimed to detect both typical and atypical events by modeling physiological data, helping identify patterns that represent normal daily behaviors as well as deviations caused by stress and anxiety.
4. Higher-order networks [22] were used to model temporal dependencies using ensemble learning frameworks, integrating multiple ML models to predict physical, psychological, and job performance variables. These models leveraged time-dependent patterns in physiological signals by incorporating lagged data.
5. Conceptual frameworks such as dynamical systems theory [40] and chaos theory [57] have been applied to capture temporal relationships. One study used a dynamical systems model with linear regression to identify daily patterns of emotional self-regulation and stress spillover in health care professionals, effectively capturing recurring cycles. In another study that used chaos theory, phase space reconstruction was applied to transform low-dimensional data into high-dimensional data by combining stress levels from multiple days as output variables. This approach enabled the model to account for previous days’ stress levels, ensuring that similar stress levels appeared closer in the reconstructed space. Additionally, integrating chaos theory with LSTM models that incorporated lagged values improved performance compared to using either chaos theory or LSTM models with lagged values independently.

### Multiple Modalities

The feasibility of using all training modalities is often limited in real-world scenarios due to the restricted availability of sensors in wearables. To address this, one study focused on reducing the number of modalities during testing while incorporating more during training [46]. This was achieved using a technique called knowledge distillation, implemented as the “More to Less” framework. Knowledge distillation involves transferring knowledge from a stronger network to a weaker one by using classifier networks from different modalities. During training, the model used all available modalities, with each having its own classifier network. These classifiers exchanged knowledge through adaptive mechanisms defined in the “More to Less” framework, allowing each classifier to learn not only from its own modality but also from those performing better. As a result, testing was conducted with fewer modalities, as the network had already developed robust representations during training. This approach enhanced generalization by leveraging information from multiple modalities, even when fewer input modalities were available during testing.

### Bias Mitigation

Bias in relation to demographic groupings was addressed in one reviewed study [56]. In that study, an LSTM model was used by incorporating multitask learning, which included both the protected attribute and the target variable. This approach aimed to mitigate bias related to the protected attribute. To achieve this, different loss weights were assigned. For example, if the target variable (eg, anxiety) is given a weight of 4.5 and the protected attribute (eg, gender) a weight of 0.5, the problem is treated as a multitask learning scenario. Each task has a separate loss function, ensuring that the nonprotected label, such as anxiety in this case, is prioritized.

### Context Awareness

Contextual information is widely recognized as crucial in studies aimed at identifying the causes or triggers of stress, as learning context helps improve model accuracy. Various types of contextual data considered in these studies are outlined below:

1. One study compared 2 methods of context collection: ML-derived data, such as sleep and activity, versus self-reported data collected through EMA [44]. The results showed that ML-derived context significantly improved model performance over self-reported data.
2. Another study focused on key stressors among employees working from home, highlighting the role of environmental factors as contextual elements affecting stress and well-being [35]. This approach highlighted the importance of both personal and environmental context in remote work settings.
3. A separate set of studies integrated circadian cycles as a contextual factor for predicting wellness indicators—such as positive affect, negative affect, and life satisfaction—by analyzing data such as audio features, heart rate, sleep patterns, activity levels, and location [49,53].
4. Various models have been used in these studies, including ensemble methods such as gradient-boosted trees (eg, CatBoost), RF, logistic regression (LR), and linear regression.

### Addressing Limited Supply of Training Data

Given the complexity of data collection in the naturalistic setting, there is a limited supply of high-quality datasets, along with challenges in capturing ground truth. To address those challenges, a study [54] explored zero-shot learning within a meta-learning framework. This framework incorporated various base learners, including RF, AdaBoost (AB), gradient boosting (GB), KNN, and LR. The approach enabled the model to learn from diverse tasks, allowing it to quickly adapt to new, unseen tasks with minimal additional training. By training on different tasks and populations, the model developed effective initialization weights, improving its ability to make accurate predictions on unseen data.

### Use of LLM in Stress Detection

Recently, with advancements in AI, emerging technologies such as LLMs have been developed. These models are pretrained on vast amounts of text and can be applied to a wide range of tasks. One study in the review used LLMs along with wearable sensor data through structured prompts that incorporated user context, health knowledge, and temporal information [59]. The study focused on three approaches:

1. Zero-shot prompting: in this approach, the model received prompts without any prior examples. Models pretrained on task-specific data, such as medical or sensor data, performed better; for instance, Asclepius outperformed larger models.
2. Few-shot prompting: a small number of examples were included in the prompt to provide context for the model. This resulted in improved performance in larger models such as GPT-3.5 (OpenAI) and GPT-4 (OpenAI).
3. Fine-tuning: this method involves adjusting some or all parameters of a pretrained model using a dataset specific to the target task. HealAlpaca achieved the highest accuracy in this category.

Overall, few-shot prompting outperformed zero-shot prompting, while fine-tuning proved to be the most effective approach.

### Analysis of Model Performance

In Table 3, we presented a detailed analysis of model performance and extracted key evaluation metrics, such as *F*_1_-score, accuracy, precision, recall, and AUC for classification tasks, and MSE for regression tasks. We reported the best-performing model in each study. Where applicable, we also documented the corresponding model configurations and comparison baselines. It should be noted that data leakage considerations are only relevant for models designed to predict momentary stress labels. Six studies [22,33,34,39,49,53] fall outside this scope, including (1) statistical models (often linear or linear mixed-effects models) that fit the entire dataset to examine coefficient significance or overall goodness-of-fit; and (2) models predicting stable psychological constructs derived from one-time baseline surveys, where the unit of analysis is the individual rather than the event or time point. We identified a total of 13 studies [30,32,35,36,41,42,45,47,54-56,58,59] that had potential data leakage issues out of 28 applicable studies [19-21,30-32,35-38,40-48,50-52,54-59].

There are a few caveats in interpreting performance measures:

1. Substantial heterogeneity exists across datasets and problem formulations (eg, classification vs regression, input and output window configurations, nowcasting vs forecasting, and different discretization strategies for stress labels), making direct, apples-to-apples comparisons challenging.
2. Performance reporting is not standardized across studies. Although *F*_1_-score is the most commonly reported metric for classification, several studies do not report it; in the case of class imbalance (which is often not reported), those metrics may not adequately reflect true model performance.
3. A notable proportion of studies (13 [30,32,35,36,41,42,45,47,54-56,58,59] out of 28 [46%] studies [19-21,30-32,35-38,40-48,50-52,54-59] among those applicable studies) has potential data leakage, most often due to inappropriate data-splitting strategies, which may inflate reported results. In fact, we note a few studies report unusually high performance (eg, with accuracy>85%), many of them with potential data leakage issues.
4. We note that a substantial proportion of studies (approximately 50%) do not report uncertainty quantification for performance metrics, such as CIs, SEs, SDs, or *P* values, making it difficult to determine whether observed performance differences are statistically meaningful or attributable to random variation.

There are several major findings:

1. Across studies, deep learning models achieved *F*_1_-scores ranging from 0.44 to 0.72, whereas traditional ML models exhibited a wider performance range (*F*_1_-score=0.47‐0.90), making it difficult to draw a general conclusion about the absolute superiority of deep learning approaches. A rigorous comparison would require controlled, head-to-head experiments that hold other factors, such as data modality, problem formulation, or data split, constant. As 2 of those examples, Parousidou et al [45] show that traditional ML combined with personalization can outperform deep learning, while Pimentel et al [55] demonstrate that careful feature engineering enables traditional models to achieve superior performance relative to deep learning methods.
2. We note that performance also varied by modeling strategy: personalization-based approaches reported *F*_1_-scores between 0.62 and 0.66; methods addressing limited data availability achieved *F*_1_-scores around 0.70; context-aware models showed a wide range with *F*_1_-score from 0.41 to 0.90; multimodal integration approaches reported an *F*_1_-score of 0.72; and models accounting for temporal dependencies exhibited wide variability (*F*_1_-score=0.23‐0.71). Finally, studies using LLMs reported accuracy values as high as 92%, although corresponding *F*_1_-scores were not provided.

Ethical Considerations

Stress detection applications and associated wearable devices often involve the collection of fine-grained longitudinal data, as well as psychological profile information, either in raw form or inferred through computational models. Such data fall within the realm of sensitive private information and, if inadvertently disclosed, may pose significant risks to individuals. For example, employers could potentially misuse stress or negative affect profiles in human resource–related decision-making processes. In addition, weak data security policies may increase the likelihood of security breaches, which could enable the reidentification of individuals and lead to the leakage of personally identifiable information. Accordingly, research studies on stress detection, as well as the downstream applications built upon these systems, must adopt deliberate and robust measures to safeguard the entire data collection, storage, and processing pipeline. These measures are essential for providing a high level of assurance and protection to individuals, whether they participate as research subjects or engage with such systems as end users of downstream applications. Some emerging approaches may also be explored, such as federated learning, as suggested in one of the reviewed studies [59]. In this approach, raw data remain on the local device where they are recorded, thereby reducing the privacy risks associated with centralized cloud-based data storage and processing.

Equally important is the need for transparent and meaningful informed consent procedures. Data owners should be clearly informed about what types of data are being collected, how the data will be processed and analyzed, who will have access to the data, how long the data will be retained, and whether the data may be reused for secondary research purposes or commercial applications. Such transparency is critical for establishing trust and supporting individuals’ autonomy in making informed decisions regarding participation. In addition, participants should be made aware of potential risks, including the possibility of reidentification or unintended disclosure of sensitive psychological or behavioral information. As noted, among the 14 datasets used across the reviewed studies, some based on primary data collection and others relying on secondary datasets, only 11 explicitly acknowledged obtaining proper institutional review board (IRB) approval, whereas the remaining 3 studies did not report such approval [19,20,32]. This observation highlights the continuing need for stronger ethical oversight and clearer reporting practices in stress detection research involving wearable and behavioral data.

### Model Card for Wearable-Based Stress Modeling

In this section, we propose a model card framework tailored for wearable-based stress modeling, adapted from the general model card template introduced by prior work [23] in the ML community. The specific focus is on models that enable the momentary estimation of current stress or to forecast stress in the future. The original template includes sections such as model details, intended use, factors, metrics, evaluation data, training data, quantitative analysis, ethical considerations, and caveats and recommendations. Given the unique characteristics of wearable-based stress modeling, particularly the reliance on longitudinal, in-the-wild biosignal data collection and self-reported ground truth, we reorganize and customize the model card into two core components: (1) dataset and (2) modeling decisions. This structure foregrounds the data provenance and methodological choices that most strongly influence model validity, transparency, and reproducibility. A detailed model card for wearables, including its components, dimensions, and reporting requirements, is presented in Table 5 below.

**Table 5.** Proposed model card reporting template for wearable-based stress detection models.

Component and dimension	Reporting requirements
Modeling decisions	
Model metadata	Model creators and affiliated organization Model version, date, citation, and license Point of contacts and link to code repository if applicable
Dataset	
Participant characteristics and context	Participant demographics (eg, age, gender, and occupation) and their distributions Sample size and duration and frequency of data collection Geographic locations of participants Naturalistic context: description of participants’ typical daily life (eg, work schedules and routines) and common stressors relevant to the population under study
Wearable devices and signals	Wearable devices used and placement Biosignals captured (eg, heart rate, HRV^a^, EDA^b^, and accelerometry) and sampling frequency
Ground truth annotation	Target variables (eg, perceived stress) and how they are captured EMA^c^ protocol details, including prompt frequency and scheduling strategy Response window and compliance requirements Survey instrument used Descriptive statistics of target variables, including distributions across demographic subgroups, where applicable
Metadata	Dataset versioning, documentation, point of contact, citations, licenses, and so on and any relevant contextual metadata (eg, time zones, device firmware, and protocol deviations)
Modeling decisions	
Data preparation	Preprocessing steps, including handling of missing data, signal cleaning, normalization, multimodal data synchronization, and identification of subgroups
Problem formulation	Task definition (eg, classification vs regression) Temporal framing Task specification Nowcasting versus forecasting input and output window configurations
Experimental design	Modeling setup and validation strategy Data splitting procedures (training, validation, and test) Assessment of potential data leakage and use mitigation strategies such as subject-level data-split
Modeling approach	Provide detailed configuration of the model, including those do not perform well Document hyperparameter tuning strategy and process Report a variety of performance metrics, including uncertainty measures such as CIs, SE, or *P* value from statistical significance test Report baseline model performance (including default model) for comparison. Decision thresholds (for classification models), if applicable
Dataset	
Ethical considerations	Ethical approval (eg, IRB^d^ and participant consent procedures) Recruitment and sampling strategy, and discuss potential of sampling bias Privacy protection strategy, eg, anonymization
Modeling decisions	
Caveats and recommendations	Justification of key modeling choices and the specific challenges they are intended to address Discussion on factors influencing performance, including reporting of disaggregated performance across relevant subgroups where applicable Discussion on sampling bias and generalization limitations, especially when transferring models across populations, contexts, or devices Other known limitations, appropriate use cases, and guidance for interpretation

a HRV: heart rate variability.

b EDA: electrodermal activity.

c EMA: ecological momentary assessment.

d IRB: institutional review board.

## Discussion

### Principal Findings

Stress detection has become increasingly important due to the rising prevalence of stress in individuals’ daily lives. Advances in wearable technologies have opened new opportunities for detecting stress in real-world settings. This scoping review aims to examine the existing literature on the use of ML applied to data collected from wearables in naturalistic environments for stress detection. In this section, we summarize the principal findings, the identified research gaps, and potential avenues for further investigation, while acknowledging the limitations of this review.

This review identified a lack of standardization in model reporting, which is one of the biggest barriers to advancing the field beyond existing research, and the generalizability of stress detection in naturalistic settings. This is due to substantial heterogeneity in how problem specifications are defined, with no standard format for model reporting, which limits benchmarking across current studies. The reviewed studies differed considerably in the wearables used, ground-truth labeling strategies, input-output window definitions, and ML approaches. We also identified methodological gaps, including potential data leakage, inconsistent reporting of performance metrics, limited handling of class imbalance, and relatively little explicit attention to motion artifacts. In addition, we noted that the reviewed studies addressed a range of challenges, though not uniformly, including personalization, temporal modeling, bias mitigation, modality-related concerns, challenges specific to in-the-wild settings, and the use of LLMs. These findings also motivated the proposed model card specification to improve comparability, transparency, and reproducibility across studies in the future.

### Methodological Rigor

In our analysis of model frameworks, characteristics, and performance summarized in Tables 2 and 3, we observed that 13 [30,32,35,36,41,42,45,47,54-56,58,59] out of 28 (46%) applicable studies [19-21,30-32,35-38,40-48,50-52,54-59], specifically, those that developed ML models to predict momentary stress had potential data leakage issues. These issues primarily arise from ignoring the grouped structure of the data, where multiple observations or episodes are contributed by the same participant. When training, validation, and test splits are performed at the observation level rather than the participant level, data points from the same individual may appear in both the training and evaluation sets. This overlap can result in unintended information leakage from training to test data, thereby artificially inflating model performance. A commonly recommended practice to mitigate this issue is to perform data splitting at the subject level, ensuring that all observations from a given participant are assigned to a single split. Given that a substantial proportion of the reviewed studies exhibit this potential methodological flaw, the performance metrics reported in Table 3 should be interpreted with caution, as they may overestimate true generalization performance.

Another issue identified in our analysis of model performance is that a large proportion of studies do not explicitly report baseline model performance. This omission is particularly problematic in the presence of class imbalance, where commonly reported metrics such as accuracy may be misleading. For example, a naïve classifier that always predicts the majority class may achieve deceptively high accuracy, despite providing little meaningful predictive value. Moreover, performance metrics that are more robust to class imbalance, such as the AUC, are rarely reported. While some studies attempt to address this issue by using alternative measures, including balanced accuracy (BACC) or Matthews correlation coefficient (MCC), such practices remain the exception rather than the norm. In addition, we observe that a substantial proportion of studies do not report uncertainty quantification for performance metrics, such as CIs or SDs. The absence of these measures complicates performance comparison across models or approaches, as observed differences may simply reflect random variation rather than meaningful performance gains.

To address these methodological gaps, we explicitly incorporate these considerations into our proposed model card specification, with the goal of encouraging future research to adopt more rigorous experimental designs, performance measures, and reporting practices.

### Standardization and Benchmarking

The complexity and richness of data collected from wearables and other devices in real-world settings create ample opportunities to explore a wide range of ML frameworks. While this flexibility allows researchers to pose a variety of research questions and explore various kinds of models, it also poses significant challenges when comparing the utility and performance of different models in tasks such as stress detection and estimation. As demonstrated in our analysis, the lack of standardization in reporting often makes it difficult to extract key specific details about ML modeling decisions, thus limiting the ability to compare model effectiveness across studies. This lack of standard and benchmarking is likely to slow progress in developing robust ML models for stress detection.

Future research could focus on the establishment of benchmark datasets and standardized tasks to enable meaningful comparisons. Additionally, the research community would benefit from adopting consistent reporting standards that ensure critical components of ML frameworks are clearly documented. For example, approaches such as model cards [23] promote transparency and reproducibility. There should also be a stronger emphasis on model quality control, including the adoption of best practices in experimental design to mitigate issues such as data leakage, which may contribute to the inflation of reported model performance. In this paper, we proposed a model card solution specifically tailored to modeling stress in the wild using wearable data, based on our analysis and understanding of the critical issues and related best practices related to modeling decisions, performance analysis, and reporting.

### Longitudinal View of Stress: From Nowcasting to Forecasting

A distinguishing feature of real-life stress studies is the ability to examine stress over extended periods and explore its interactions with contextual factors. Despite these opportunities, we observed that most studies adopt a short-term approach to stress detection, where the input and output windows are closely aligned in time. Only a few recent studies have begun to explore forecasting methods that account for long-term temporal trends in psychological and physiological states.

Among the 4 forecasting-oriented papers, 2 adopt a conventional ML pipeline focused on next-day stress prediction. Paraschou et al [47] report an accuracy of 92.3% from a GB ML model, although the evaluation may be subject to data leakage concerns. Another study [50] systematically explores a wide range of input and output window configurations and finds that LSTM-based models generally achieve the strongest performance. In particular, the highest predictive accuracy is obtained when using data from the preceding 15 days (ie, input window=15 days) to predict next-day stress (ie, output window=1 day), although XGBoost (eXtreme Gradient Boosting) models show competitive performance under several configurations. In contrast, the remaining 2 papers adopt dynamic systems modeling approaches [40,57]. Hadjiantonis et al [40] fit a linear dynamical system model that characterizes next-day changes in emotional arousal as a linear function of the current day’s perceived stress and emotional arousal, with model parameters estimated via linear regression. Li et al [57], however, model day-to-day perceived stress as a nonlinear dynamical system. The study follows a 2-step approach: first, chaos theory is used to identify the most reliable prediction horizon, which shows that an input window of 19 days maximizes predictability for forecasting stress over the subsequent 35 days. Using phase-space embedding and deep neural networks, the proposed method achieves accuracies of 74.4% for binary stress classification and 69.23% for 3-level stress classification under a person-wise 80/20 split.

We encourage future research to broaden the scope of stress modeling beyond momentary detection, incorporating longitudinal perspectives that capture the ebb and flow of stress over time. Such studies could also investigate the interplay between stress and mediating factors such as sleep, as well as coping strategies such as meditation, to provide a more comprehensive understanding of stress dynamics in daily life. Our review suggests that methodological innovations adopting multivariate and longitudinal perspectives are still emerging. Traditional ML pipelines provide a reasonable starting point by framing stress forecasting as a mapping between multimodal signal streams within predefined input windows and corresponding output windows. However, much of the existing work relies on black-box models that offer limited insight into the underlying mechanisms of stress-related dynamics. A complementary line of research draws on dynamical systems–inspired approaches, including both linear and nonlinear models. Within these frameworks, prediction targets often extend beyond a single future time point to a prediction horizon spanning multiple days or weeks. This shift enables more meaningful and theoretically grounded questions to be addressed, such as the evolution trajectory and long-term trends of stress dynamics over time. Chaos theory–based approaches, in particular, provide a useful lens for understanding the limits of predictability in forecasting future stress trajectories. Li et al [57] represent a promising first step in this direction, and future work could further advance this line of inquiry by incorporating richer, multivariate biosignals from wearable devices in both the input and output spaces.

For both ML-based and dynamical systems–based models, a key challenge in fitting highly complex models lies in data sparsity and limited transparency, as these approaches often require large amounts of high-quality longitudinal data while offering limited interpretability. One promising direction for addressing these challenges is the systematic integration of domain knowledge, such as insights from psychological theory, through principled frameworks such as neurosymbolic systems [60,61], which combine data-driven learning with theory-informed constraints and representations. Advancing such approaches will likely require close multidisciplinary collaboration between computing researchers and domain experts in fields such as psychology and health care.

### Addressing Modeling Challenges in the Wild

Data collection in real-life settings, where participants are moving, poses significant challenges to the validity of physiological signals due to motion artifacts. Additionally, it remains unclear to what extent models trained on laboratory-controlled datasets generalize to real-world scenarios. Our analysis found that only a few studies explicitly acknowledge and address these challenges. To address motion artifacts, a study [42] proposed subband HRV features in addition to traditional HRV benchmark features (time-domain and frequency-domain features). The proposed features were obtained by splitting the HRV tachogram into low-frequency (LF) and high-frequency (HF) bands and analyzing each band separately. From these bands, the authors computed nonlinear features, such as transfer entropy between LF and HF components, as well as spectral descriptor features, such as centroid, spread, skewness, kurtosis, crest, and spectral entropy. The study found that its Fuse-All model, which combined the traditional benchmark HRV features with the proposed subband features, gave better performance than the benchmark features alone. Another study [36] took a deliberate approach to compare the models developed in the lab versus those in the wild. They used a commercial off-the-shelf heart rate monitor in both laboratory and real-world settings on the same cohort of participants to ensure comparability. Stress labels collected in the wild were obtained through EMA-based self-reports. The results showed that the best-performing pipeline involved removing extreme values through trimming (ie, outlier removal), followed by data standardization using z-score normalization (trim_zscore). Furthermore, excluding periods of high physical activity further improved performance in real-world settings.

### Model Transparency

In addition to evaluating model performance using common metrics such as accuracy, an equally important aspect of model quality is transparency. Transparent models not only allow for better diagnosis and refinement but also create opportunities for interdisciplinary collaboration with experts from fields such as psychology, who bring deep knowledge of stress and its mechanisms. However, many current modeling approaches rely on black-box methods—particularly those based on deep learning and, more recently, generative AI. While these models may improve performance in certain cases, they also make interpreting results more challenging [59]. Future research should explore modeling approaches that achieve a better balance between transparency and accuracy. By integrating domain knowledge from psychology and physiology, such models could not only predict stress but also explain why certain predictions are made—uncovering not just correlational relationships, but potentially causal ones that offer insights for designing effective stress interventions. Additionally, developing models and systems that facilitate clear communication of model decisions to end users could open a new line of inquiry focused on ensuring human-in-the-loop approaches in stress modeling as explored in the study by Paraschou [47].

### LLMs in Wearable-Based Stress Detection

With the rise of LLMs, there is increasing interest in using them for mental health prediction tasks such as stress, anxiety, depression, and sleep disorders. One study included in this review demonstrated promising predictive performance using both larger LLMs, such as GPT-3.5 and GPT-4, and a smaller health-focused fine-tuned model, HealthAlpaca. The study suggested that performance depends not only on model size but also on prompting strategy, contextual information, and domain-specific fine-tuning, with smaller fine-tuned models performing comparably to or better than larger general-purpose LLMs. It also showed that the performance of larger models can be improved by providing examples through few-shot prompting or by adding contextual information to zero-shot prompts. However, several limitations remain. Wearable time-series data are high-dimensional, nonlinear, and continuous, making them more challenging for LLMs than conventional language inputs. In addition, the validity and interpretability of LLM-based predictions remain important concerns due to the lack of standardized evaluation benchmarks for nonlinguistic wearable data. These models also have substantial data and computational demands, as well as the risk of false-positive or hallucinated outputs in health-related settings. Future research should therefore examine explainability, validity, reliability, and whether LLM-based approaches provide meaningful advantages over more conventional models in wearable-based stress prediction.

### Data Quality and Validity Considerations 

Our review highlights several recurring data quality and validity challenges in wearable-based stress studies conducted in naturalistic settings. In particular, field-collected datasets are often shaped by study design constraints, resulting in nonuniform data distributions, limited sample sizes, short observation periods, and skewed participant characteristics (eg, gender, age, and other demographic attributes). These factors collectively constrain population representativeness and limit the external validity and generalizability of reported findings. In contrast, studies that rely on secondary or publicly available large datasets typically benefit from longer monitoring durations and more heterogeneous participant pools. While such datasets may offer improved statistical power and demographic coverage, they also introduce trade-offs, including reduced control over data collection protocols, labeling procedures, and contextual fidelity. Taken together, these differences underscore the importance of explicitly reporting dataset characteristics and carefully considering how data provenance and study design choices shape model validity and interpretability in real-world stress modeling. In our proposed model solution, we explicitly include sections on dataset characteristics to encourage robust study design, as well as transparency and standardization in reporting.

### Limitations

Limitations of this scoping review include the challenge of ensuring that all relevant studies have been included and none have been overlooked due to language constraints, as well as the exclusion of unpublished or ongoing studies. Additionally, we focused on a limited range of databases, which restricts the scope of the studies retrieved. The search criteria and query string used may also limit the outcomes of the scoping review. Rather than implementing full parallel double-screening of all records, we adopted a variant of the screening approach in which one researcher conducted the initial screening, followed by a review by a second reviewer. As a result, interrater reliability metrics, such as Cohen κ or percentage agreement, were not calculated. The absence of formal double-screening and corresponding reliability statistics may limit the reproducibility of the screening process.

### Conclusion

Wearable devices offer valuable opportunities to measure and understand how stress manifests in real-world settings. ML and other advanced computational and statistical methods have increasingly been adopted to develop models that detect, characterize, and understand stress and related psychological constructs from physiological signals captured by wearables, as well as from self-reported data collected at increasingly fine temporal granularity using methods such as EMA. In this focused review, we examined recent studies that apply ML and advanced computational methods to model stress using data from wearable devices in the wild. This review provides an in-depth assessment of current modeling practices and reported performance in this domain and adds to existing reviews by focusing specifically on problem formulation, detailed analysis of model performance, and methodological rigor. In particular, we highlight key problem formulation decisions, such as input and output window configuration, and make the distinction between nowcasting and forecasting tasks, and conduct a detailed analysis of model performance across varied modeling approaches. Through this analysis, we observe a few general patterns of model performance as a function of modeling approaches. We also identify important methodological issues, such as potential data leakage, which may threaten the validity of reported results and inflate reported model performance. We also noted a lack of standardization in datasets, task definitions, and reporting practices, making cross-study comparisons challenging and potentially slowing progress in wearable-based stress detection research. In response to these findings, we propose a model card framework to guide modeling decisions, experimental design, and reporting practices, with the goal of promoting methodological rigor, enhancing transparency, and reproducibility. We hope this review serves as a roadmap for new researchers entering the field, while also offering a framework for researchers working on similar problems to share findings and collaboratively advance the field as a community.

## Supplementary material

10.2196/76632 Multimedia Appendix 1 Search strategy and study selection for PubMed (PRISMA-ScR Item 8).

10.2196/76632 Multimedia Appendix 2 Dataset characteristics (XLSX file, 9 KB).

10.2196/76632 Multimedia Appendix 3 Machine learning framework (XLSX file, 16 KB).

10.2196/76632 Checklist 1 PRISMA-ScR checklist.

## Abbreviations

AB: AdaBoost
AUC: area under the receiver operating characteristic curve
BACC: balanced accuracy
BI-LSTM: bidirectional long short-term memory network
CNN: convolutional neural network
EMA: ecological momentary assessment
FFN: feedforward neural network
GB: gradient boosting
GMM: Gaussian mixture model
HF: high frequency
IRB: institutional review board
IRF: impulse response function
KNN: k-nearest neighbors
LF: low frequency
LLM: large language model
LR: logistic regression
LSTM: long short-term memory
MCC: Matthews correlation coefficient
ML: machine learning
MSE: mean-squared error
PANAS: Positive and Negative Affect Schedule
PCA: principal component analysis
PRISMA: Preferred Reporting Items for Systematic Reviews and Meta-Analyses
PRISMA-ScR: Preferred Reporting Items for Systematic Reviews and Meta-Analyses extension for Scoping Reviews
PSS: Perceived Stress Scale
RF: random forest
RNN: recurrent neural network
S-STAI: Short State-Trait Anxiety Inventory
SNN: Siamese neural network
SOM: self-organizing map
STAI: State-Trait Anxiety Inventory
STAI: State-Trait Anxiety Inventory
SVM: support vector machine
VAR: vector autoregression
XGBoost: eXtreme Gradient Boosting
