# Supplementary material for: Machine Learning Frameworks for Wearable-Based Stress Modeling in Naturalistic Settings: Scoping Review
Source: JMIR Mhealth Uhealth. 2026 Jul 31;14:e76632. doi: 10.2196/76632 (PMC13427063; doi:10.2196/76632)
Supplement: Multimedia Appendix 1 [file mhealth-v14-e76632-s001.docx]

**Multimedia Appendix 1**

## Search Strategy and Study Selection for PubMed (PRISMA-ScR Item 8)

## **Information Source**

Database searched: PubMed

Date of search: April 30, 2024

## **Search Strategy**

The following search strategy was applied in PubMed:

((stress detection) AND (wearables)) AND (physiology)

## **Filters Applied**

• Publication date: January 1, 2017 – April 30, 2024
• Language: English

## **Search Results and Study Selection**

Records retrieved from PubMed: n = 100
Records retained after title screening: n = 42
Records retained after abstract screening: n = 23
Full-text articles assessed for eligibility: n = 23
Full-text articles included in the scoping review: n = 4

## **Notes on Reporting**

This supplementary file presents the full electronic search string and the limits applied in PubMed, in accordance with Item 8 of the PRISMA Extension for Scoping Reviews (PRISMA-ScR). It is provided to ensure transparency and reproducibility of the search process. The study selection counts reported here correspond to the PRISMA-ScR flowchart presented in the main manuscript.
